# Serum human epididymis protein 4 level as a predictor of clinical worsening in idiopathic pulmonary arterial hypertension: a pilot study

**DOI:** 10.1186/s12872-020-01461-w

**Published:** 2020-04-15

**Authors:** Qi Jin, Yi Tang, Zhihong Liu, Wenlin Xie, Qin Luo, Zhihui Zhao, Qing Zhao, Zhiwei Huang, Xue Yu, Lu Yan, Changming Xiong, Xinhai Ni, Yinkun Yan

**Affiliations:** 1 State Key Laboratory of Cardiovascular Disease, Center for Pulmonary Vascular Diseases, Fuwai Hospital, National Center for Cardiovascular Diseases, Chinese Academy of Medical Sciences and Peking Union Medical College, 167 Beilishi Road, Xicheng District, Beijing, 100037 China; 2 Department of Cardiology, Hunan Provincial People’s Hospital, The First Affiliated Hospital of Hunan Normal University, The College of Clinical Medicine of Hunan Normal University, Changsha, China; 3 Department of Pathology, The Seventh Affiliated Hospital of Sun Yat-sen University, Shenzhen, China; 4 Department of Epidemiology, Capital institute of Pediatrics, Beijing, China

**Keywords:** Human epididymis protein 4, Idiopathic pulmonary arterial hypertension, Cardiac function, Predictor, Clinical worsening

## Abstract

**Background:**

Human epididymis protein 4 (HE4) was proved to be a novel biomarker for left heart failure. The purpose of this exploratory study was to evaluate the role of HE4 in patients with idiopathic pulmonary arterial hypertension (IPAH) who usually have concurrent right heart failure.

**Methods:**

55 patients with newly diagnosed IPAH were continuously enrolled and serum HE4 levels were assessed at baseline. All patients were followed up from the date of blood sampling, and a composite endpoint of clinical worsening was detailedly recorded.

**Results:**

Serum levels of HE4 were significantly higher in IPAH patients than healthy controls (6.9 ± 2.2 vs 4.4 ± 0.9 ng/ml, *p* < 0.05) and increased as cardiac function deteriorated. HE4 levels correlated with endothelin-1 (*r* = 0.331, *p* < 0.01) and right atrial pressure (*r* = 0.30, *p* < 0.03). After a mean follow-up of 20 ± 10 months, 13 patients experienced clinical worsening. Receiver operating characteristic analysis showed that HE4 levels > 6.5 ng/ml discriminated clinical worsening with a sensitivity of 92.31% and a specificity of 59.52% (area under the curve [AUC] = 0.81). Multivariate Cox regression analysis demonstrated that HE4 (χ^2^: 5.10; hazard ratio [HR] = 1.26; 95% confidence interval: 1.03 to 1.55, *p* < 0.02) and pulmonary vascular resistance (χ ^2^: 4.19; HR = 1.14; 95% confidence interval: 1.00–1.29, *p* < 0.04) were independently predictive of clinical worsening. Patients with HE4 > 6.5 ng/ml had a worse 2-year survival rate than those with HE4 ≤ 6.5 ng/ml (58.9% vs 96.2%, *p* < 0.001).

**Conclusions:**

Serum levels of HE4 were elevated in IPAH patients and correlated with disease severity. HE4 was an independent predictor of clinical worsening in IPAH patients.

## Background

Pulmonary arterial hypertension (PAH) is a life-threatening condition characterized by extensive narrowing and obliteration of the pulmonary vasculature, leading to increased pulmonary vascular resistance (PVR) and subsequent right heart failure and terminal death. The US-REVEAL registry displayed a low 5-year survival rate of 61.2% in patients with newly diagnosed PAH [1]. Therefore, early diagnosis and prognostic stratification are important for the optimal therapeutic strategies for PAH patients. Circulating biomarkers, such as N-terminal prohormone brain-type natriuretic peptide (NT-proBNP), endothelin (ET)-1 and growth differentiation factor-15, have been proposed as parameters for diagnostic and prognostic evaluation. Nevertheless, these biomarkers do not fully reflect all features of the complex pathophysiology of PAH [2]. Therefore, the quest for new biomarkers is highly desirable.

Human epididymis protein 4 (HE4) was originally identified as a secreted protein in the human epididymis [3]. It was mildly expressed in other organs such as kidney and respiratory tract [4]. HE4 could suppress the activity of multiple proteases such as serine proteases and matrix metalloproteinases, specifically inhibit degradation of type I collagen, and mediate kidney fibrosis [5]. Interestingly, HE4 correlated with acute and chronic left heart failure severity and predicted adverse outcomes in heart failure, and the strong correlation between HE4 and galectin-3 or fibroblast growth factor 23 demonstrated that cardiac fibrosis was putatively involved in the pathogenic process [6, 7]. However, the role of HE4 has not been evaluated in patients with idiopathic pulmonary arterial hypertension (IPAH), who usually die of right heart failure characterized by right ventricular fibrosis and remodeling. Therefore, this pilot study aimed to assess the prognostic value of HE4 in IPAH patients.

## Methods

### Study population

Between February 2013 and November 2015, 55 adult patients with newly diagnosed IPAH admitted to Fuwai Hospital were enrolled in the study. IPAH was defined according to the guideline [8]. Patients with the following conditions were excluded due to potential interference in HE4 concentrations: 1). current smoker, 2). recent pregnancy, 3). cancer, and 4). chronic renal failure [9]. The control group was age- and sex-matched healthy subjects without the above conditions. This study was conducted in compliance with the Declaration of Helsinki and was approved by the institutional review board of Fuwai Hospital. All patients signed written informed consent.

### Clinical assessment and follow-up

Diagnostic right heart catheterization (RHC) was performed using standard hemodynamic measurements at baseline as previously described [10]. Basic demographics such as body mass index (BMI) and World Health Organization functional class (WHO-FC) and biochemical tests were collected at baseline. Serum NT-proBNP and ET-1 concentrations were measured using commercially available ELISA kits (Biomedica GmbH, Vienna, Austria).

Patients were followed-up every 3 months for 1 year and every 6 months later after discharge. The composite end point was defined as the time from the date of blood sampling to the first clinical worsening (CW) event, including all-cause mortality, lung transplantation, hospitalization for PAH worsening, need for intravenous epoprostenol therapy, and interventional procedures (balloon atrial septostomy), as previously reported [11]. The follow-up time was calculated from the time of blood sampling to May 1, 2016.

### HE4 measurement

Peripheral venous blood samples were collected the next day morning after RHC. Blood samples for the measurement of HE4 were tubed with ethylenediaminetetraacetic acid anticoagulant and centrifuged for 15 min at 3000 rpm within 30 min of collection. Serum samples were then stored at − 80 °C for further analysis. Hemolyzed specimens were kicked out before experiments. Serum levels of HE4 were measured using the Quantikine® human HE4/ Whey acidic protein four-disulfide core domain 2 (WFDC2) immunoassay (R&D systems, Inc., USA & Canada). The minimum detectable dose of human HE4 ranged from 2.44 to 32.2 pg/ml. The intra- and inter-assay coefficients of variation were less than 3.5 and 5.9% respectively. All measurements were performed by an investigator blinded to the clinical data.

### Statistical analysis

The data were presented as mean ± standard deviation for continuous variables or as percentages for categorical variables. Statistical differences between two groups were analyzed using an independent Student’s t test for normally distributed variables and Mann–Whitney U test for non-normally distributed variables. The data were visually inspected for normality and outliers using scatter plots. Chi-square test was used for categorical variables. Correlations between HE4 and other variables were explored using Pearson or Spearman correlation coefficients as appropriate. The optimal cutoff value of HE4 was determined by receiver operating characteristic (ROC) curve analysis. Univariate Cox regression analysis was used to explore the predictive value of each variable for clinical worsening, and then a forward stepwise multivariate Cox regression model was further used to evaluate variables with *p* < 0.05. Kaplan–Meier plots with log-rank tests illustrated outcomes of patients grouped by the optimal cutoff value of HE4. Statistical analyses were performed using SPSS version 19.0 (SPSS, Inc). A two-sided *p*-value < 0.05 was considered statistically significant.

## Results

### Baseline characteristics

Fifty-five IPAH patients (31 ± 9 years, 83.6% female) were enrolled in our study. The numbers of patients in WHO-FC I/II/III/IV were 3/25/26/1 respectively. Their mean PVR was 10.8 ± 4.7 Wood units with a mean cardiac index (CI) of 2.8 ± 0.9 L·min^− 1^·m^− 2^. 50 (90.9%) patients were treated with monotherapy (39 with phosphodiesterase type 5 inhibitors, 9 with endothelin receptor antagonists and 2 with prostacyclin), 2 patients received combination therapy and three were treated with calcium channel blockers. Their baseline characteristics were summarized in Table 1.

**Table 1:** Baseline characteristics between CW and Non-CW IPAH patients

Variables	Total (*n* = 55)	CW (*n* = 13)	Non-CW (*n* = 42)	*P* Value
Age, years	31 ± 9	34 ± 8	30 ± 10	0.11
Female, n (%)	46(83.6)	11(84.6)	35(83.3)	1.0
BMI, kg/m^2^	22.6 ± 3.0	21.7 ± 2.5	22.9 ± 3.1	0.18
WHO-FC III-IV, n (%)	27(49.1)	9(69.2)	18(42.8)	0.12
Laboratory tests				
Creatinine, μmol/l	71.3 ± 15.2	74.1 ± 18.8	70.5 ± 14.0	0.46
Uric acid, μmol/l	443.2 ± 131.0	469.7 ± 172.1	435.0 ± 116.8	0.41
HsCRP, mg/l	1.7 ± 1.6	1.5 ± 1.5	1.8 ± 1.6	0.44
NT-proBNP, pg/ml	1181.8 ± 1102.3	1389.4 ± 1505.2	1117.6 ± 958.8	0.84
ET-1, fmol/ml	0.5 ± 0.6	0.7 ± 0.8	0.5 ± 0.5	0.62
HE4, ng/ml	6.9 ± 2.2	8.8 ± 2.5	6.3 ± 1.7	0.001
Hemodynamics				
RAP, mmHg	4.9 ± 4.7	8.0 ± 5.1	4.0 ± 4.2	0.01
mPAP, mmHg	50.1 ± 10.6	51.5 ± 9.0	49.7 ± 11.0	0.59
PCWP, mmHg	6.6 ± 2.8	7.2 ± 2.9	6.4 ± 2.8	0.30
PVR, Wood units	10.8 ± 4.7	12.3 ± 5.3	10.3 ± 4.5	0.19
CI, L·min^−1^·m^−2^	2.8 ± 0.9	2.5 ± 0.6	2.9 ± 0.9	0.23
SvO_2_, %	70.5 ± 7.3	68.7 ± 6.7	71.0 ± 7.4	0.47

*CW* Clinical worsening, *IPAH* Idiopathic pulmonary arterial hypertension, *BMI* Body mass index, *WHO-FC* World Health Organization functional class, *HsCRP* High sensitive C-reactive protein, *NT-proBNP* N-terminal prohormone brain natriuretic peptide, *ET-1* Endothelin-1, *HE4* Human epididymis protein 4, *RAP* Right atrial pressure, *mPAP* Mean pulmonary arterial pressure, *PCWP* Pulmonary capillary wedge pressure, *PVR* Pulmonary vascular resistance, *CI* Cardiac index, *SvO2* Mixed venous oxygen saturation

### Comparison between groups

**Fig. 1:**
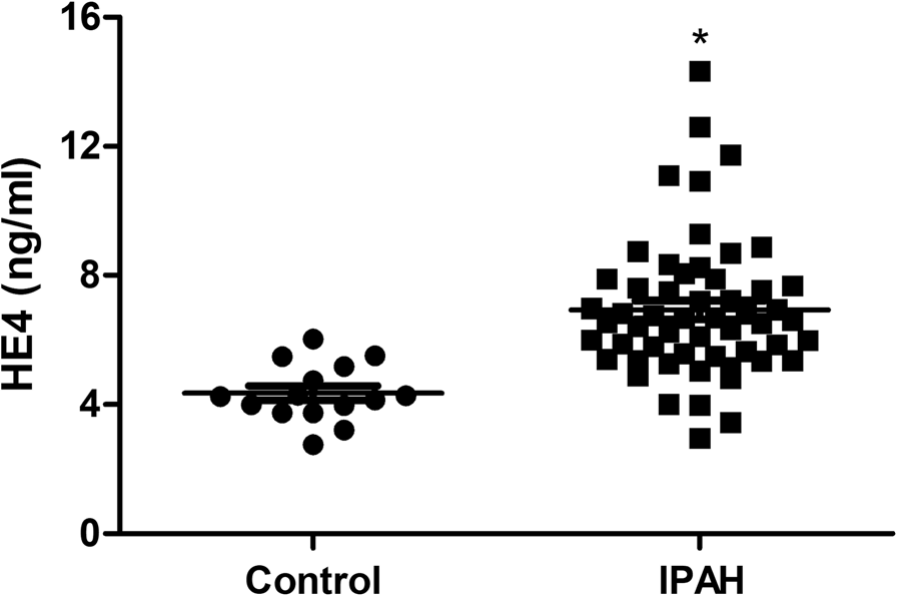
Serum HE4 levels in IPAH patients and control subjects. ^*^p<0.05. *HE4* human epididymis protein 4, *IPAH* idiopathic pulmonary arterial hypertension

15 healthy volunteers were enrolled in our study. Age and sex did not differ between IPAH patients and healthy controls. Interestingly, HE4 levels were notably higher among IPAH patients compared to those of control subjects (6.9 ± 2.2 vs 4.4 ± 0.9 ng/ml, *p*<0.001, Fig. 1). In addition, HE4 levels in WHO-FC III/IV patients were significantly increased compared to those in WHO-FC I/II (7.7 ± 2.7 vs 6.2 ± 1.2 ng/ml, *p*<0.009). HE4 levels correlated positively with ET-1 (*r* = 0.331, *p* < 0.01) and right atrial pressure (RAP) (*r* = 0.30, *p*<0.03). However, no significant correlations were observed between HE4 and other variables.

The mean follow-up duration was 20 ± 10 months, and no patients were lost to follow-up. Four patients died (three for right heart failure and one for sudden death), one underwent lung transplantation and eight worsened. Comparison between the parameters of CW and non-CW patients was shown in Table 1, and no differences were observed in sex, age, and BMI between the two groups. Only HE4 and RAP in CW patients were significantly higher than those in non-CW patients, and none of other clinical variables were significantly different.

### HE4 and clinical outcomes

**Fig. 2:**
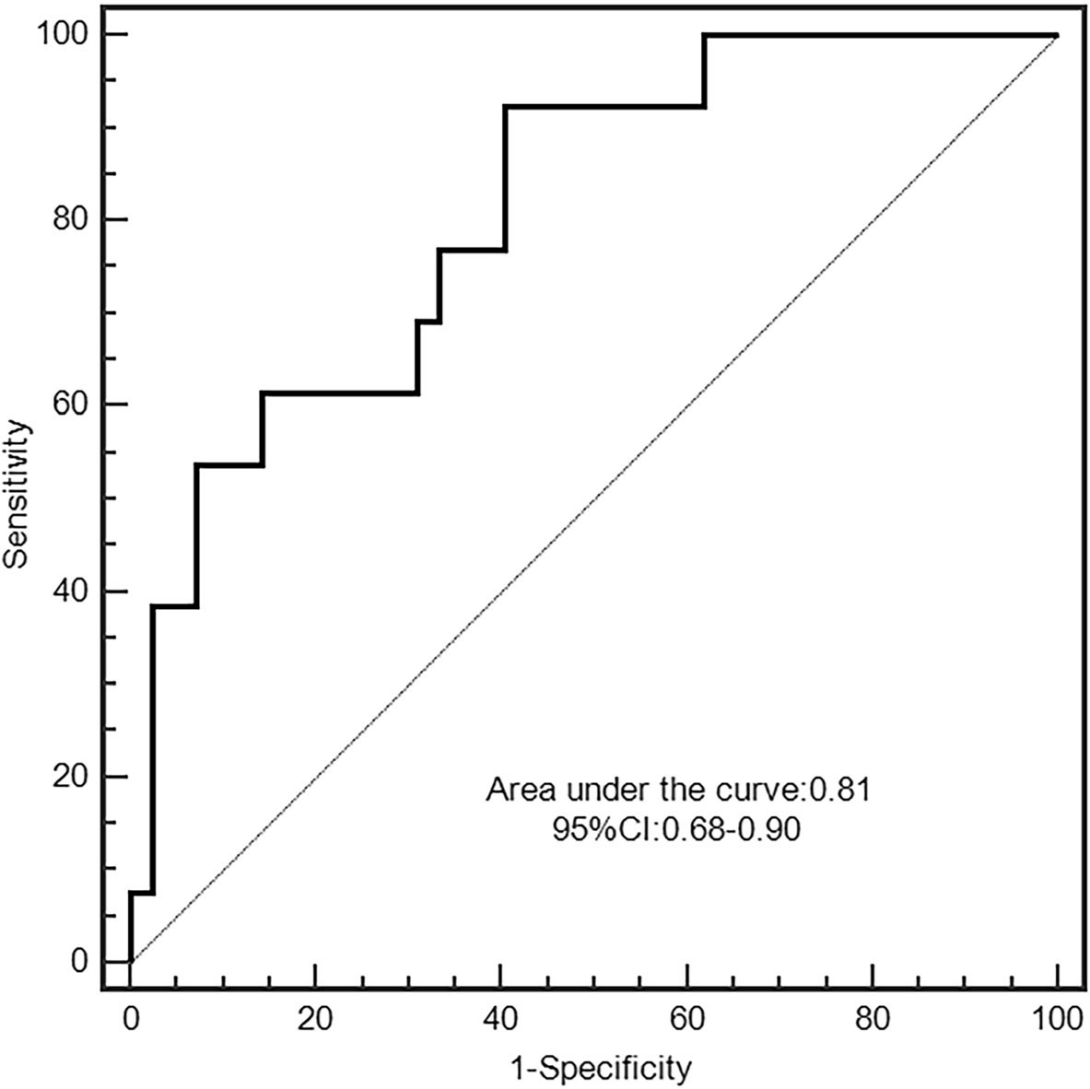
ROC curve showing the sensitivity and specificity of HE4 to predict clinical worsening. *ROC* receiver operating characteristic, *HE4* human epididymis protein 4

The ROC analysis showed HE4 levels > 6.5 ng/ml predicted clinical worsening with a sensitivity of 92.3%, a specificity of 59.5%, and the area under the curve (AUC) of 0.81 (*p*<0.001, Fig. 2). Furthermore, HE4 outperformed other prognostic markers such as CI (AUC = 0.61, *p* = 0.03), PVR (AUC = 0.60, *p* = 0.03) and NT-proBNP (AUC = 0.52, *p* = 0.001).

In univariate analysis, HE4 (hazard ratio [HR] = 1.30, 95% confidence interval [CI]: 1.06–1.59, *p* = 0.01), WHO-FC (HR = 4.05, 95% CI: 1.18–13.91, *p* = 0.02), RAP (HR = 1.15, 95% CI: 1.03–1.29, *p* = 0.02), PVR (HR = 1.15, 95% CI: 1.02–1.29, *p* = 0.03) and CI (HR = 0.36, 95% CI: 0.14–0.93, *p* = 0.04) were all significant predictors of CW. Multivariable forward stepwise Cox analysis was performed including HE4, WHO-FC, RAP, PVR and CI, showing HE4 (χ^2^: 5.10; HR = 1.26; 95% CI: 1.03–1.55, *p* < 0.02) and PVR (χ^2^: 4.19; HR = 1.14; 95% CI: 1.00–1.29, *p* < 0.04) could independently predict clinical worsening.

**Fig. 3:**
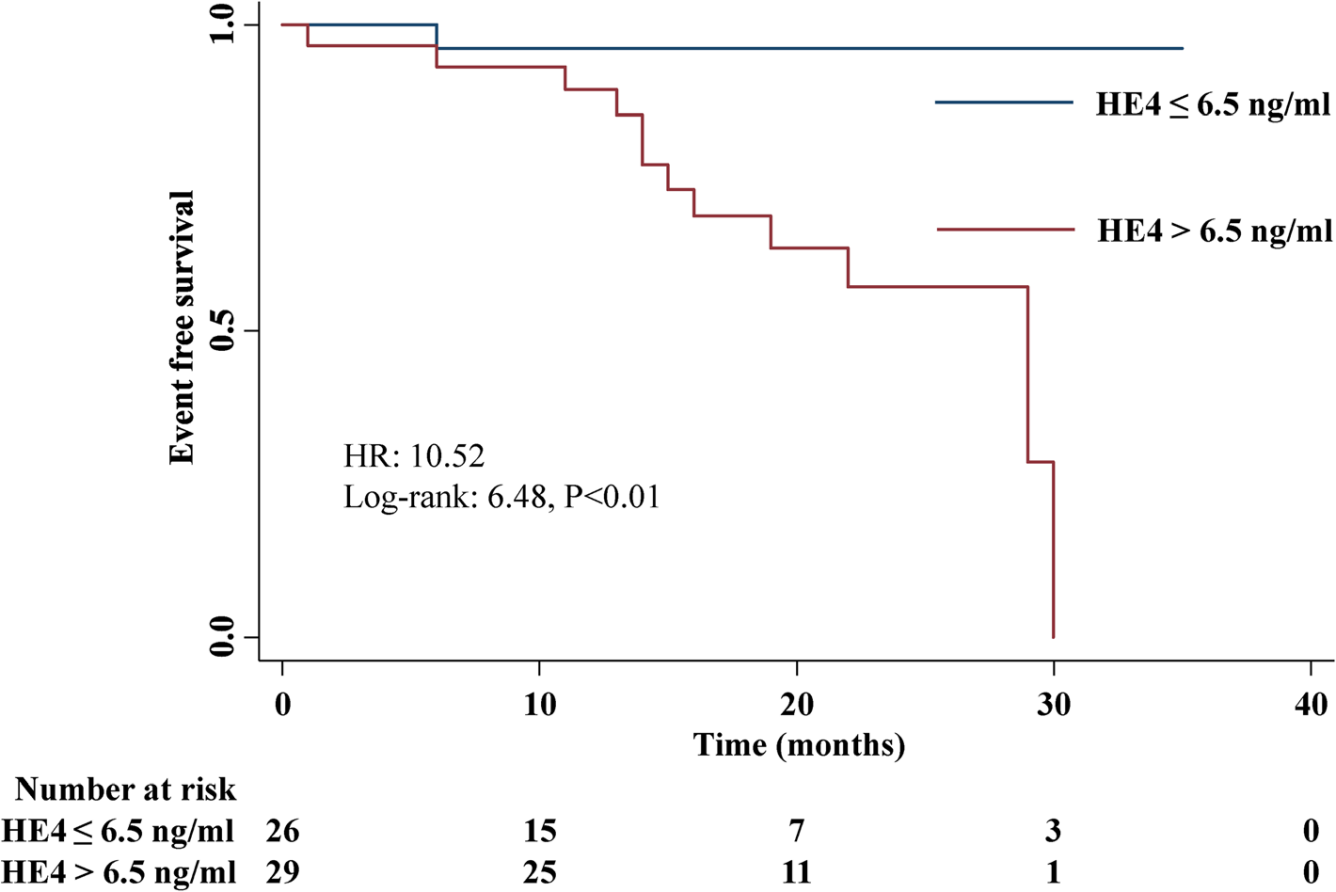
Kaplan–Meier analysis of serum HE4 levels for clinical worsening. *HE4* human epididymis protein 4, *HR* hazard ratio

Kaplan-Meier survival curves were shown in Fig. 3. Patients with HE4 > 6.5 ng/ml had a worse 2-year survival rate than those with HE4 ≤ 6.5 ng/ml (58.9% vs 96.2%, *p* < 0.001). The unadjusted HR was 10.52 (95% CI: 1.33–83.65), and the HR after adjustment for age, sex, and BMI was 10.74 (95% CI: 1.26–9.48).

## Discussion

To the best of our knowledge, this is the first study to prove that elevated serum HE4 levels could serve as a novel biomarker for IPAH patients. Herein, we revealed that HE4 enhanced as cardiac function deteriorated, and correlated with ET-1 and RAP. Moreover, our present study demonstrated HE4 was a powerful independent prognostic factor for clinical worsening in IPAH patients. The significant increase of serum HE4 level may indicate a poor prognosis, and early intensive targeted therapy for patients with high HE4 level may improve their clinical outcomes.

HE4, also known as WFDC2, was mildly to moderately expressed in epididymis, kidney, respiratory tract, and salivary glands [4]. Several studies reported serum HE4 was overexpressed in ovarian cancer and lung cancer patients [12, 13], and it might also play a role during innate immune defense and tumorigenesis [14, 15]. Fibroblast-derived HE4 could mediate kidney fibrosis via suppressing the activity of multiple proteases, such as serine proteases and matrix metalloproteinases, which could be inhibited by HE4 neutralizing antibodies in mouse models [5]. Additionally, HE4 levels were elevated in patients suffering from chronic kidney disease and left heart failure [6, 7, 16], denoting that HE4 could potentially play an essential role in renal and cardiac fibrosis.

Our study illustrated that HE4 levels were higher in IPAH patients than healthy controls, and its levels increased as cardiac function deteriorated, which was consistent with previous studies among left heart failure patients [6, 7]. Furthermore, HE4 had a weak but significant positive correlation with RAP and ET-1, and elevated RAP indicates right ventricular dysfunction, predicts poor outcome in PAH patients [17], and is usually triggered by cardiac fibrosis [18]. Elevated ET-1 levels could also independently predict clinical worsening in IPAH patients treated with Bosentan, and ET-1 played a crucial role in vascular and tissue fibrosis [19, 20]. In addition, HE4 had a strong positive correlation with galectin-3, a biomarker of cardiac fibrosis, indicating that HE4 might function in cardiac fibrosis [7]. These evidences might establish a link among HE4, RAP and ET-1. However, HE4 was not correlated with NT-proBNP and creatinine, which might be ascribed to the small sample size and limited number of patients in WHO-FC I or IV.

IPAH is characterized by pulmonary vascular remodeling, and IPAH patients generally die from right heart failure. Extracellular matrix protein collagen and fibrosis are the key factors involved in pulmonary vascular remodeling and right heart failure [21, 22]. Although we suspected that HE4 could potentially play a crucial role in cardiac fibrosis, the exact mechanism between HE4 and right heart failure as well as pulmonary vascular remodeling remains largely unknown.

Our study has several limitations. First, the relatively small sample size and few clinical events might create potential bias, as this is only a preliminary study to investigate the possible role of HE4 in pulmonary vascular disease and right heart failure, the population size is being expanded for further validation. Second, HE4 levels were detected only at baseline, and changes in HE4 levels after treatment were unknown. Third, we did not measure levels of galectin-3 and matrix metalloproteinase 2/9, which might provide additional mechanistic insights into the link between HE4 and IPAH. Therefore, further large sample studies are needed to investigate the role of HE4 in IPAH patients.

## Conclusion

Serum levels of HE4 were elevated in patients with IPAH and could independently predict clinical worsening. HE4 might be a novel biomarker for IPAH.

## Data Availability

The datasets used and analyzed during the present study are available from the corresponding author on reasonable request.

## Abbreviations

PAH: Pulmonary arterial hypertension
PVR: Pulmonary vascular resistance
NT-proBNP: N-terminal prohormone brain-type natriuretic peptide
ET-1: Endothelin-1
HE4: Human epididymis protein 4
IPAH: Idiopathic pulmonary arterial hypertension
RHC: Right heart catheterization
BMI: Body mass index
WHO-FC: World health organization functional class
CW: Clinical worsening
WFDC2: Whey acidic protein four-disulfide core domain 2
ROC: Receiver operating characteristic
CI: Cardiac index
RAP: Right atrial pressure
AUC: Area under the curve
